# Effect of tissue-grouped regulatory variants associated to type 2 diabetes in related secondary outcomes

**DOI:** 10.1038/s41598-023-30369-6

**Published:** 2023-03-02

**Authors:** Daiane Hemerich, Roelof A. J. Smit, Michael Preuss, Lauren Stalbow, Sander W. van der Laan, Folkert W. Asselbergs, Jessica van Setten, Vinicius Tragante

**Affiliations:** 1 The Charles Bronfman Institute for Personalized Medicine, Icahn School of Medicine at Mount Sinai, New York, NY USA; 2 Central Diagnostics Laboratory, Division Laboratories, Pharmacy, and Biomedical Genetics, University Medical Center Utrecht, Utrecht University, Utrecht, The Netherlands; 3 Department of Cardiology, Amsterdam University Medical Centers, University of Amsterdam, Amsterdam, The Netherlands; 4 Health Data Research UK and Institute of Health Informatics, University College London, London, UK; 5 Department of Cardiology, UMC Utrecht, Utrecht University, Utrecht, The Netherlands

**Keywords:** Medical genomics, Personalized medicine

## Abstract

Genome-wide association studies have identified over five hundred loci that contribute to variation in type 2 diabetes (T2D), an established risk factor for many diseases. However, the mechanisms and extent through which these loci contribute to subsequent outcomes remain elusive. We hypothesized that combinations of T2D-associated variants acting on tissue-specific regulatory elements might account for greater risk for tissue-specific outcomes, leading to diversity in T2D disease progression. We searched for T2D-associated variants acting on regulatory elements and expression quantitative trait loci (eQTLs) in nine tissues. We used T2D tissue-grouped variant sets as genetic instruments to conduct 2-Sample Mendelian Randomization (MR) in ten related outcomes whose risk is increased by T2D using the FinnGen cohort. We performed PheWAS analysis to investigate whether the T2D tissue-grouped variant sets had specific predicted disease signatures. We identified an average of 176 variants acting in nine tissues implicated in T2D, and an average of 30 variants acting on regulatory elements that are unique to the nine tissues of interest. In 2-Sample MR analyses, all subsets of regulatory variants acting in different tissues were associated with increased risk of the ten secondary outcomes studied on similar levels. No tissue-grouped variant set was associated with an outcome significantly more than other tissue-grouped variant sets. We did not identify different disease progression profiles based on tissue-specific regulatory and transcriptome information. Bigger sample sizes and other layers of regulatory information in critical tissues may help identify subsets of T2D variants that are implicated in certain secondary outcomes, uncovering system-specific disease progression.

## Introduction

**Figure 1:**
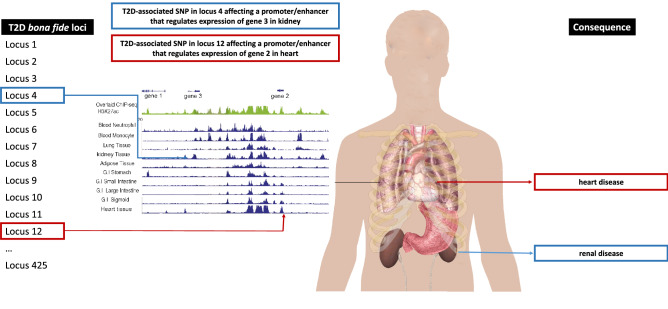
Hypothetical tissue-specific consequences of T2D variants. A number of *bona fide* SNPs have been associated with T2D risk. A subset of these SNPs overlaps enhancers, promoters and eQTLs in different tissues. The variant might affect the regulatory element it overlaps, and consequently the expression of the gene affected by this regulatory element. Carriers of particular sets of tissue-specific regulatory SNPs might manifest consequences of T2D in a different way, with different progression and outcome profiles.

Type 2 diabetes mellitus (T2D) has an estimated prevalence of 10% in the United States and is on the rise^1,2^, leading to increased risk of premature death^3^, prolonged hyperglycemia from insulin resistance and relative insulin deficiency^4^, and numerous micro- and macrovascular complications^5^. These complications affect several organs and tissues, causing e.g. heart damage, eye problems, and nerve disease. Even though there are over 500 independent genetic variants associated with T2D^6–8^, there is little understanding of their pathophysiology leading to T2D itself and to secondary outcomes. As with other complex traits, most T2D-associated variants are located within non-coding regions of the genome, and might interrupt the action of regulatory elements crucial in relevant tissues^9^. Several studies point to an enrichment of T2D-associated variants in tissues such as pancreas, adipose, skeletal muscle, liver, arteries, kidney and the heart^8,10,11^. These are also the tissues affected by secondary outcomes related to diabetes, such as heart disease, nephropathy or peripheral artery disease. Genes controlled by regulatory elements affected by DNA variation act in different pathways in these tissues, and disturbance in gene expression is often reflected in a variety of outcomes for which T2D is a risk factor. Thus, T2D-associated variants altering expression of genes in the heart may more likely affect disease progression through heart-mediated processes rather than kidney-mediated processes. It follows that, while all patients are initially diagnosed with T2D, some patients may develop coronary artery disease while others may suffer kidney failure (Fig. 1).

**Figure 2:**
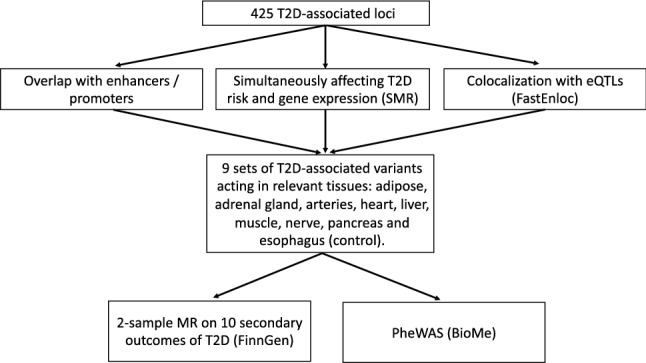
Overview of the approach. We selected variants from 425 T2D-associated loci acting on enhancers, promoters and eQTLs of nine tissues relevant for T2D and its secondary outcomes. We used the nine tissue-grouped variant sets as input for downstream analyses to test their effects on ten secondary outcomes related to T2D: 2-sample MR and PheWAS.

We aimed at identifying T2D progression profiles and their impact in different secondary outcomes whose risk is increased by T2D. To this end, we studied the effect of the combination of sets of T2D-associated variants that show tissue-specificity based on epigenetic markers and expression quantitative trait loci (eQTL) in secondary outcomes related to T2D (Fig. 2). We identified subsets of T2D-associated variants or single-nucleotide polymorphisms (SNPs) acting in regulatory elements and expression quantitative trait loci (eQTL) of relevant tissues, and assessed whether each subset had an increased risk to develop secondary outcomes related to T2D, in order to unravel tissue-specific genetic profiles that could increase risk of an outcome affecting tissues of interest.

## Results

### Overview of the approach

We identified subsets of T2D-associated variants acting in regulatory elements or influencing expression of genes in tissues relevant to T2D and secondary outcomes related to T2D (Fig. 2). We then associated these variant sets to T2D-related outcomes through both 2-sample Mendelian randomization (MR) and PheWAS, in order to investigate for differential (causal) effects..

### T2D-associated variants are enriched in pancreas, heart, eye, liver and kidney

We applied LD score regression (LDSC) in epigenetic data from the Epigenome Integration across Multiple Annotation Projects (Epimap), replicating the cell-type enrichment of T2D-associated variants previously observed in^8,10^ (“Methods”). LDSC can be used to test whether a particular genome annotation, such as histone marks, capture more heritability than expected by chance. The strongest enrichment was in pancreas (p = 8.7 × 10^−4^), followed by heart (p = 0.01), endometrial adenocarcinoma (p = 0.012), Multi-Potent Progenitor (MPP) cells (p = 0.014), eye (p = 0.016), liver (p = 0.017) and kidney (p = 0.032) (Supplemental Table 1). Previous studies also highlight the involvement of adipose tissue^8^ and skeletal muscle^12^. Due to the involvement of pancreas, heart, eye, liver, kidney, skeletal muscle and adipose tissue in secondary outcomes related to T2D, we selected these tissues for downstream analysis. Despite arteries, nerve and adrenal gland not showing significant results in our enrichment analyses, we included these tissues due to their known involvement in subsequent outcomes of T2D^13–16^. We used esophagus as a control tissue for downstream analysis, a tissue ranked one of the least significant p-values in the LDSC analysis and is not known to be involved in secondary outcomes related to T2D.

### Identification of sets of variants acting on regulatory elements in relevant tissues

We used three methods to identify subsets of T2D variants acting in tissues involved in secondary outcomes related to T2D: overlap with enhancers and promoters, Summary-based Mendelian Randomization (SMR)^17^ and Fast Enrichment Estimation Aided Colocalization Analysis FastENLOC^18,19^.

#### Overlap with enhancers and promoters

We identified high confidence enhancers in nine tissues of interest, namely adipose, adrenal gland, arteries, heart, kidney, liver, muscle, nerve, pancreas and esophagus (control) (“Methods”). Briefly, we used as criteria for a confidence enhancer to be present in at least 2/3 of all datasets of its category in adult tissue present on Epimap database^20^. According to these criteria, no datasets of adult kidney were available on Epimap^20^, so we excluded kidney from downstream analysis. We identified an average of 28,047 enhancers and 19,483 promoters in the datasets included (Supplemental Table 2). We further identified enhancers and promoters that are unique to each of the nine tissues of interest. An average of 2371 unique enhancers and 603 unique promoters were identified.

Next, we overlapped all 425 T2D-associated SNPs and their non-coding proxies in high linkage disequilibrium (LD) (N = 14,007) with these high confidence enhancers and promoters, as well as the unique enhancers and promoters (“Methods”).

#### Summary-based Mendelian Randomization (SMR)

We ran SMR^17^ to identify variants that affect both gene expression and T2D risk. An average of 75 non-independent variants per tissue passed the SMR and HEIDI tests thresholds (“Methods”). We selected significant SMR results unique to each of the nine tissues of interest, in comparison with all datasets from the Genotype-Tissue Expression (GTEx) project.

#### FastENLOC

We used fastENLOC^18,19^ to identify T2D-associated variants that colocalize with eQTLs in the tissues of interest. An average of six non-independent variants per tissue passed the colocalization threshold (“Methods”). We selected unique fastENLOC results as those significant results unique to each of the nine tissues of interest, in relation to all datasets from GTEx.

After performing all three analyses for identification of subsets of variants acting on tissues relevant for T2D and its secondary outcomes, we further narrowed down these subsets to only independent variants (“Methods”). The final subsets of T2D variants had on average 176 SNPs, while subsets of T2D variants acting on unique regulatory elements had on average 30 SNPs (Table 1). These were used as input/instruments on 2-sample MR analyses and PheWAS analyses.

**Table 1:** Number of SNPs and their F-statistics (median, 25th and 75th percentiles) in each tissue-grouped variant set, using all regulatory elements and tissue-specific (unique) regulatory elements.

Tissue	All regulatory elements		Tissue-specific (unique) regulatory elements	
	N	F-statistics median (IQR)	N	F-statistics median (IQR)
Adipose	196	39.8 (31.4–64.6)	45	51.9 (36.3–71.9)
Adrenal gland	140	40 (31.7–60.6)	13	50.6 (38.8–71.2)
Aorta/arteries	209	39.3(31–59.2)	65	34.6 (31.2–56.9)
Esophagus	195	39.8 (32–60.4)	64	43.4 (33.8–56.8)
Heart	186	39.2 (31.6–63.7)	52	37.4 (26.7–69.6)
Liver	167	39.8 (31.5–64.9)	44	36.9 (29.7–64)
Muscle	165	39.4 (30.9–59)	60	39.7 (29.7–53.7)
Nerve	172	38.9 (31.3–58.6)	17	36.5 (31.7–64.7)
Pancreas	150	39.4 (31.3–58.9)	24	35.4 (29.7–44.8)

*IQR* interquartile range.

### No causal relation between tissue-grouped variant sets and T2D secondary outcome

We ran MR analyses to assess the association of each tissue-grouped variant set as genetic instruments to secondary outcomes related to T2D (“Methods”). Briefly, MR is a method that uses genetic variants to estimate causal effects between the exposure and outcome under a set of assumptions, such as independence of confounding factors^21^. MR-analysis was performed using an inverse-variance weighted (IVW) linear regression, with instrument-outcome associations as dependent variable, instrument-exposure associations as independent variable, and with the intercept constrained to zero (“Methods”). Considering all T2D genetic instruments (425 lead variants identified in^7^), an increase in T2D risk was associated with an increased risk of all outcomes tested, apart from the control esophagitis (Supplemental Fig. 1). In the tissue-grouped MR analyses, for all outcomes tested except chronic kidney disease (CKD) and the control outcome esophagitis, all tissue-grouped variant sets showed to increase risk of secondary outcomes related to T2D, including the set of our control tissue, esophagus (Supplemental Fig. 1). These results were consistent both when using tissue-grouped variant sets of variants overlapping all regulatory elements in tissues of interest, as well as regulatory elements unique to each of the tissues of interest. Due to the lower power of tissue-grouped sets of variants acting in unique regulatory elements, their confidence intervals were much wider than the much bigger sets of SNPs overlapping all regulatory elements in tissues of interest. In the analysis including variants overlapping all regulatory elements, as expected, the outcome with most risk increase was T2D itself (highest T2D odds ratio (OR) 2.44, 95% (confidence interval (CI) 2.29–2.60) in adrenal gland, 2.44 (95% CI 2.30–2.59) in nerve). The other outcomes (apart from the control esophagitis) had ORs between 1.04 (95% CI 0.93–1.16) for CKD in the pancreas subset, and 2.07 (95% CI 1.68–2.53) for diabetic neuropathy in the adipose subset (Supplemental Fig. 1, Supplemental Table 3). However, we did not observe an instance in which a tissue-grouped variant set increased risk of a secondary outcome of T2D more than others. We also performed three complementary analyses which relax the assumption of no horizontal pleiotropy amongst the genetic variants. First, MR-Egger regression, of which the intercept formally tests for the presence of unbalanced horizontal pleiotropy, and the slope reflects the causal effect estimate after adjusting for this pleiotropy by adding an intercept to the IVW method^22^. We also applied weighted median-based estimator^23^ and the weighted mode-based estimator^24^, which respectively use the weighted median of, and the highest density of, the ratio estimates across the individual instruments as estimate of the true causal effect. Sensitivity analyses using MR Egger, Weighted Median and Weighted Mode were largely consistent with IVW results, and similarly did not provide evidence for heterogeneity across variant sets (Supplemental Table 3).

### Phenome-wide analyses of tissue-specific genetic risk scores (GRSs)

Finally, we took a disease-agnostic approach and tested the association of the GRS of each tissue-grouped variant set (i.e., SNPs overlapping with all regulatory elements in the nine tissues of interest) with phenotypes in a PheWAS analysis^31,32^ (“Methods”). The only phenotype to pass Bonferroni correction was T2D and its variations, such as T2D with renal manifestations or T2D with peripheral circulatory disorders (Supplemental Fig. 2). When assessing results at nominal significance (p < 0.05), no tissue-grouped variant set was associated to diseases linked to both T2D and the tissue itself, such as cardiovascular diseases associated to heart-grouped or artery-grouped variants, or obesity-related diseases associated to adipose-grouped variants (Supplemental Fig. 2).

## Discussion

We hypothesized that subsets of variants associated with T2D could have tissue-specific effects, and therefore would influence the emergence of specific secondary outcomes. Similar to previous analyses, we observed an enrichment of T2D variants in pancreas, heart, eye, liver and kidney^8,10^. We obtained subsets of T2D-associated regulatory variants in these tissues when available, and also others previously implicated in T2D etiology (adipose, adrenal gland, skeletal muscle, arteries and nerve).

We used the selected tissue-grouped variant sets in two analyses to assess their association with secondary outcomes related to T2D: 2-sample MR and PheWAS. T2D loci found through GWAS might speak primarily to the development of T2D (and the tissues important to developing T2D) rather than tissue-specific downstream consequences of T2D. We did not observe, in any of the analyses carried out, that a tissue-grouped variant set increases risk of any particular outcome more than other tissue-grouped variant sets, or has a specific disease signature.

The identification of T2D-associated variants acting in regulatory elements and gene expression is limited to the databases of regulatory elements and gene expression available. Despite the great number of regulatory elements identified by Epimap^20^, we still do not have the full catalog of regulatory elements in all human tissues and cells. Recent large-scale common and rare GWAS suggest that substantially larger association studies are needed to identify most T2D loci in the population^25^. Similarly, larger datasets capturing the regulatory landscape of the human genome in relevant tissues are needed to help explain T2D-associated loci, and this work can be extended and applied to more high powered eQTL databases^26,27^ and more recently published atlas of relevant single-cell epigenomes. *Currently available single-cell epigenomic data include T2D-relevant tissue such as coronary artery *^28^*, heart *^29^*, and pancreatic islets*^30^. An extension of this work could also benefit from a more refined selection of input variants associated to T2D, such as the fine-mapped credible sets of potential causal variants for each T2D risk locus made available by^31^. Such efforts will increase the potential of identification of causal variants acting on gene regulation, and might identify groups of T2D-associated variants that have specific effects in disease risk.

Moreover, despite being assumed that the majority of GWAS-associated loci, which are non-coding, exert small regulatory effects on the expression of genes, the majority of disease-associated genetic variants have not yet been clearly explained by current eQTL data^32–35^. Studies have found that 5–40% of trait associations co-localize with eQTLs in relevant tissues^36–40^. In fact, a study designed specifically to investigate the link between genetic association and regulatory function has failed to capture it. The authors observed that for the majority of putatively causative genes considered, no fine-mapped variants were associated with regulatory regions in relevant tissues^40^. The authors speculate that lack of statistical power could be one of the reasons, as well as the biological context – causative eQTLs may only manifest in certain developmental windows, under specific conditions, or in a crucial cell subpopulation^40^. The above may explain why another effort to identify tissue-grouped variant sets based on tissue-expression profiles has similarly failed to identify different disease risks for their tissue-grouped sets^41,42^. However, a more recent study utilizing larger gene expression datasets for brain and subcutaneous adipose tissue showed evidence that BMI-associated variants colocalizing with gene expression in brain tissue might be driving the genetically predicted effects of BMI on cardiovascular-disease endpoints, whereas adipose tissue variants might predominantly explain the effects of BMI on measures of cardiac function^43^.

Another limitation of this study is that GWAS summary statistics for the secondary outcomes related to T2D studied where all control individuals have diabetes were not available. Thus, while for example in the case of outcomes such as diabetic nephropathy it is possible that many controls had diabetes, we could not filter for those individuals specifically, and the control group may include individuals without diabetes, and a mix of type 1 and type 2 diabetes.

To conclude, our novel approach for the identification and assessment of tissue-grouped T2D-associated variants did not find evidence for significantly different causal effects in any tissue-grouped variant set that could be used for prediction of secondary outcomes related to T2D. Increasing sample sizes, both in the number of participants as the number of regulatory variants identified in each specific tissue, may overcome the limitations faced in this study. Moreover, the use of datasets at the single-cell resolution could help capture effects not observed in analysis of RNA sequencing performed on bulk tissue. As more experiments on the investigation of the regulatory landscapes in a variety of tissues are performed, the more data will be available for such integration, and the more our knowledge will increase on how regulatory variants act on specific tissues and the interplay of regulatory elements. Further investigation on tissue-specific genetic risk profiles can not only help us understand the disease mechanisms, but also build a basis for tissue-specific, genetic profile-driven therapeutics.

## Methods

### Ethical approval

All research was performed in accordance with relevant guidelines/regulations, and informed consent for sequencing, phenotype assessment, and publication of results was obtained at time of enrollment for BioMe biobank and FinnGen participants. The Coordinating Ethics Committee of the Helsinki and Uusimaa Hospital District has evaluated FinnGen, and the EU Data Protection Regulation that came into force in May 2018 has been taken into account when planning the project. Further details can be found in https://www.finngen.fi/en/code_of_conduct. BioMe Biobank was approved by the Program for the Protection of Human Subjects. Further details are located in the BioMe researcher FAQ (https://icahn.mssm.edu/research/ipm/programs/biome-biobank/researcher-faqs) and https://icahn.mssm.edu/research/pphs.

### Description of the cohorts

The Mount Sinai BioMe Biobank is an ongoing electronic health record (EHR)-linked biorepository that enrolls participants non-selectively from the Mount Sinai Health System^44^. It has included 60,000 participants from the greater New York City area since its inception in 2007. Participants are between 18 and 89 years of age and represent a broad spectrum of racial and ethnic diversity (African (24%), European (32%), Hispanic-Latino (35%) and other (9%) ancestries). At enrollment, participants consent to link their DNA and plasma samples to their de-identified EHRs. Clinical and EHR information are complemented by a detailed questionnaire that gathers demographic and lifestyle information. The median number of clinical encounters for BioMe participants is 21.

The FinnGen study utilizes samples collected by a nationwide network of Finnish biobanks. The study is based on combining genome information with digital health care data from national health registries. The R5 freeze used in this study consists of > 218,700 individuals, up to 17 M variants and > 2800 phenotypes^45^.

### Variant selection and tissue enrichment

We obtained summary statistics including 425 loci identified by a GWAS meta-analysis*,* including 21 independent (p < 5e − 8, > 500 kb and LD r^2^ < 0.05) variants identified in Europeans only, 153 novel independent SNPs identified in the transethnic meta-analysis, and 251 independent established T2D variants^8^. Full summary statistics comprise SNP, chromosome, position, effect and non-effect allele frequencies, beta, standard deviation, p-value and N. The full meta-analysis included 1.4 million participants and identified a total of 568 associations across all ancestries. We used LDSC^46^ to perform tissue enrichment analysis, integrating the full summary statistics from Vujkovic et al*.*^8^ with 806 datasets of predicted enhancers from the Epimap project^20^.

### Tissue-grouped variant sets

We selected variants acting on regulatory elements in each tissue of interest by overlapping variants with enhancers or promoters and *bona fide* eQTLs. In a secondary analysis, we identified enhancers, promoters and eQTLs that are unique to the tissues of interest, and overlapped those with T2D-associated loci. Variants passing these criteria were then grouped by tissue, and each tissue group was narrowed down to independent variants using function *clump* from PLINK v1.9^47^, parameters --clump-p1 1e-5 --clump-kb 500 --clump-r2 0.001, using 1000 Genomes phase3 Europeans as reference panel^48^. GRS was calculated using function –score from PLINK v1.9^47^, weighted by European-specific effect sizes from Vujkovic et al.^8^.

### T2D variants acting on regulatory elements

We used enhancers and promoters predicted within the scope of Epimap^20^. We downloaded chromHMM tracks on 35 tissues and cell-types from https://personal.broadinstitute.org/cboix/epimap/ChromHMM/observed_aux_18_hg19/. A list of samples used in this analysis and grouped by tissue is available on Supplemental Table 2. We included only tissues that had at least three replicates generated from adult tissues. We selected enhancer regions classified by ChromHMM as EnhA1, EnhA2, EnhG1 and EnhG2. We selected as promoters regions classified by ChromHMM as TssA, TssFlnk, TssFlnkU or TssFlnkD. We assessed how many times each enhancer and promoter appear across all datasets of each tissue, using *bedtools multiinter*^49^. We then retrieved only enhancers or promoters that appear in at least two thirds of the total number of datasets available for each tissue (Supplemental Table 2).

After building a database of high confidence enhancers and promoters in 35 tissues, we also identified regulatory elements that are unique to each tissue, using function *bedtools intersect -v*. We then overlapped T2D variants and their proxies in high LD with the full set of confidence enhancers/promoters identified in the nine tissues of interest, and the set of enhancers/promoters unique to each tissue of interest, using function *bedtools intersect*. High LD was defined as r^2^ > 0.8, retrieved using FUMA v1.3.6b^50^ and their built-in database of 10,000 randomly selected unrelated Europeans from the UKBiobank^51,52^ as reference panel. A total of 14,007 variants were intersected.

### T2D variants acting on eQTLs

We used two methods to identify T2D-associated variants influencing gene expression: SMR and colocalization with fastEnloc. SMR integrates gene expression information to pinpoint candidate causal variants by determining whether the association between an associated SNP and the phenotype is mediated through an eQTL^17^. fastENLOC is a Bayesian hierarchical colocalization method that prioritizes candidate causal variants by colocalizing associated variants and eQTLs^18^. For both analyses, we used as input data from GTEx v8^36^. Datasets included eQTL information on the tissues relevant for T2D and its secondary outcomes: adipose subcutaneous (n = 663), adipose visceral (n = 541), adrenal gland (n = 258), artery aorta (n = 432), artery coronary (n = 240), artery tibial (n = 663), heart atrial appendage (n = 429), heart left ventricle (n = 432), liver (n = 226), muscle skeletal (n = 803), nerve tibial (n = 619) and pancreas (n = 328). We used esophagus–gastroesophageal junction (n = 375), mucosa (n = 555) and esophagus muscularis (n = 515) as control tissues.

Briefly, the SMR & HEIDI approach integrates summary-level data from GWAS and eQTL studies to test if a transcript and phenotype are associated because of a shared causal variant (i.e., pleiotropy)^17^. We retrieved variants that simultaneously affect gene expression and T2D risk that passed a Bonferroni corrected p-SMR and a p-HEIDI > 0.05, as in similar studies^17^. LD data required for the HEIDI test were estimated from genotyped data from the UK Biobank (UKB) study, including 10,000 randomly selected European participants.

We applied fastENLOC^18,19^, a Bayesian hierarchical colocalization method, to assess which T2D-associated variants colocalize with eQTLs in tissues of interest. We used pre-computed GTEx v8 multi-tissue eQTL annotation available on https://github.com/xqwen/fastenloc. Variants that passed the threshold for SNP colocalization probability (SCP) > 0.1 were considered “colocalizing”.

In order to retrieve eQTLs unique to each tissue of interest, we first ran SMR and fastENLOC on all tissues available on GTEx. We then selected eQTLs passing SMR and fastENLOC significance thresholds that are unique to each of the nine tissues of interest in this study.

### Two-sample Mendelian Randomization

Previous works have described the methods for MR analysis of summary data based on two studies^53,54^. Here, we used all variants in the tissue-grouped variant sets as proposed instruments to measure their associations to ten outcomes with summary data available from the FinnGen study^45^: T2D (n total = 215,654; n cases = 35,607), diabetic nephropathy (n total = 213,746; n cases = 3,283), CKD (n total = 216,743; n cases = 3902), peripheral artery disease (PAD) (n total = 213,639; n cases = 7098), heart failure (HF) (n total = 218,208; n cases = 13,087), stroke (n total = 180,862; n cases = 18,661), myocardial infarction (MI) (n total = 200,641; n cases = 11,622), diabetic retinopathy (n total = 216,666; n cases = 14,584), diabetic neuropathy (n total = 163,616; n cases = 1415) and esophagitis (n total = 190,442; n cases = 747) as a control (case definition for all outcomes can be found on Supplementary Table 4). SNP-exposure associations were retrieved from the summary statistics from Vujkovic et al.^8^, and SNP-outcome associations come from summary statistics from Finngen^45^. All tissue-grouped variant sets were considered composed of sufficiently strong instruments based on their F-statistics, considering an F-statistic > 10 as strong enough instrument to avoid weak instrument bias^55^ (Table 1). Using fixed effects IVW analyses, we combined the effects of the individual genetic instruments to obtain a genetically determined association between exposure and outcome under the assumption of the absence of horizontal pleiotropy. Some variants from the tissue-grouped variant sets were removed from MR analyses due to being palindromic genetic instruments with intermediate allele frequencies. Estimates from the IVW analyses can be interpreted as the odds ratio for the outcome trait(s) per 2.72-fold increase in the odds of T2D (i.e., a one unit change in genetic liability to T2D on the log odds scale). We also run sensitivity analyses using methods MR Egger^22^, Weighted Median^23^ and Weighted Mode^24^. Analyses were run using the R-based package ‘TwoSampleMR’^56^.

### Phenome-Wide Association Study (PheWAS)

We used the PheWAS package in R^57^ using default settings to test for associations between our tissue-grouped variant sets and a wide range of phenotypes. We included 1039 disease outcomes in 8370 individuals of self-reported European ancestry from BioMe biobank^44^, using age, sex, body mass index (BMI) and 10 first principal components as covariates. We report the ten most significant associations. Results were corrected for multiple testing by Bonferroni test.

## Supplementary Information

Supplementary Figures.

Supplementary Tables.

## Data Availability

T2D summary statistics were downloaded from dbGaP^7^ (Study Accession: phs001672.v1.p1). All the scripts used in this project can be found at https://github.com/DaianeH/2SampleMR-T2D, together with tables containing tissue-specific T2D-associated variants used as input for PheWAS and SMR.

## Abbreviations

BMI: Body mass index
CI: Confidence interval
CKD: Chronic kidney disease
DNA: Deoxyribonucleic acid
EHR: Electronic health record
eQTLs: Expression quantitative trait loci
fastENLOC: Fast Enrichment Estimation Aided Colocalization Analysis
GRS: Genetic risk score
GTEx: Genotype-Tissue Expression
HF: Heart failure
IQR: Interquartile range
IVW: Inverse-variance-weighted
LD: Linkage disequilibrium
LDSC: LD Score regression
MI: Myocardial infarction
MPP: Multi-potent progenitor
MR: Mendelian randomization
OR: Odds ratio
PAD: Peripheral artery disease
PheWAS: Phenome-wide association studies
RNA: Ribonucleic acid
SCP: SNP colocalization probability
SMR: Summary-based Mendelian Randomization
SNP: Single-nucleotide polymorphism
T2D: Type 2 diabetes
UKB: UK Biobank
