# Supplementary material for: Effect of tissue-grouped regulatory variants associated to type 2 diabetes in related secondary outcomes
Source: Sci Rep. 2023 Mar 2;13:3579. doi: 10.1038/s41598-023-30369-6 (PMC9981672; doi:10.1038/s41598-023-30369-6)
Supplement: Supplementary file 1 — Supplementary Figures. [file 41598_2023_30369_MOESM1_ESM.docx]

**Supplemental Material**

**Supplemental Figures**

Average = 186

**
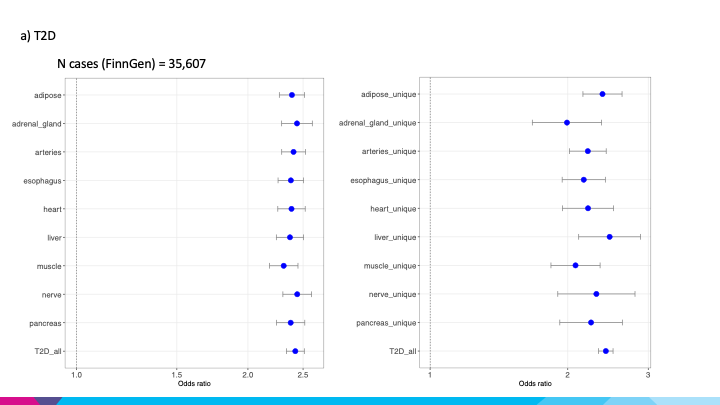
**

**
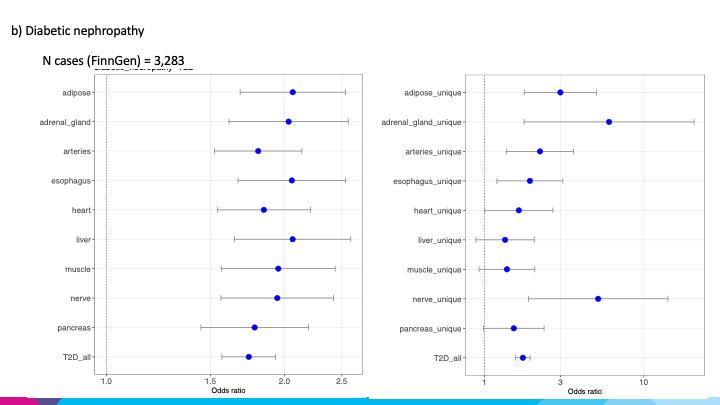
**

**
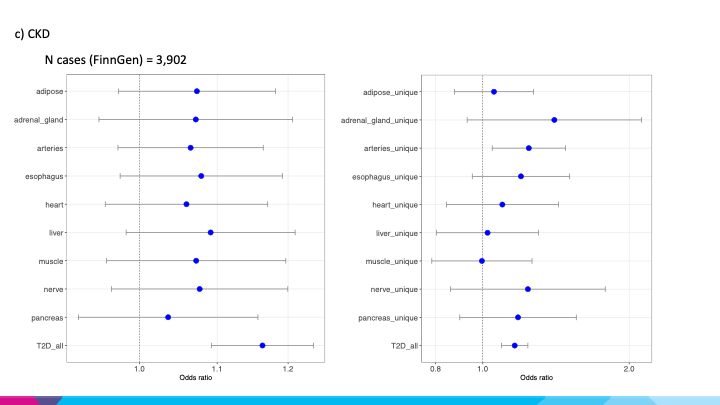
**

**
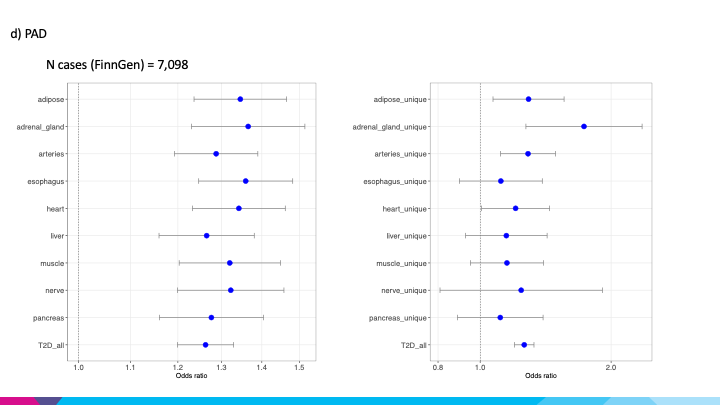
**

**
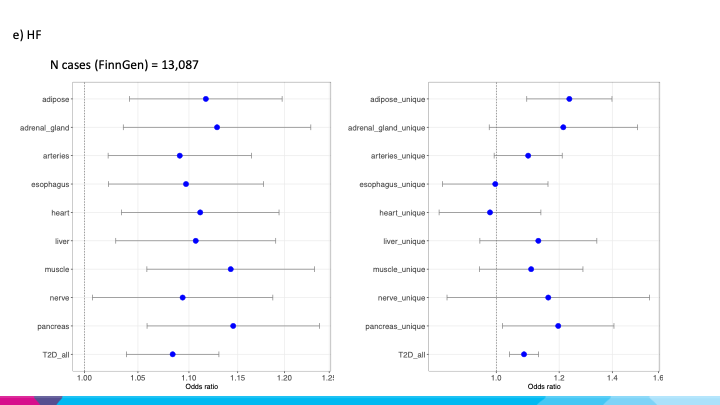
**

**
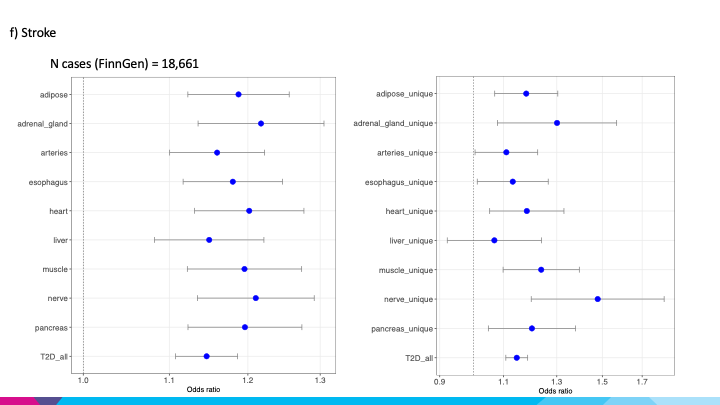
**

**
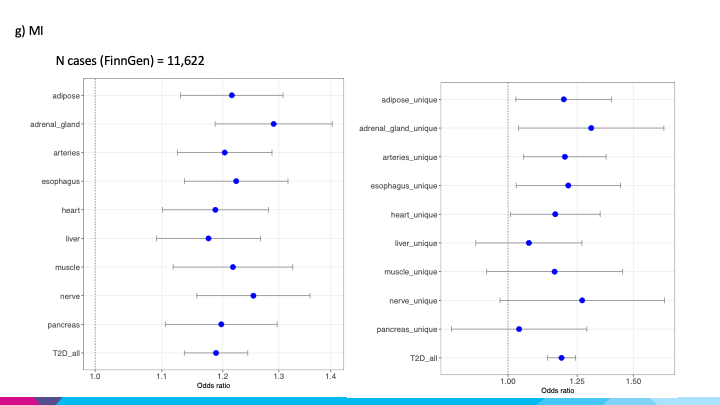
**

**
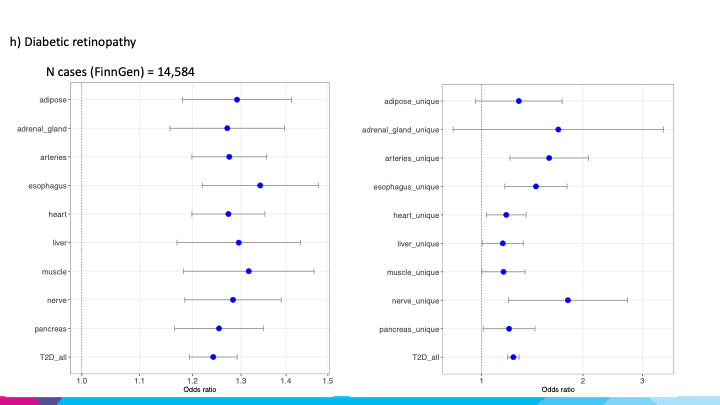
**

**
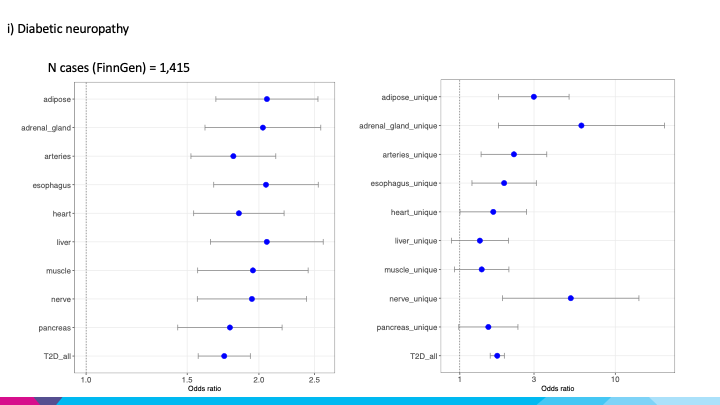
**

**
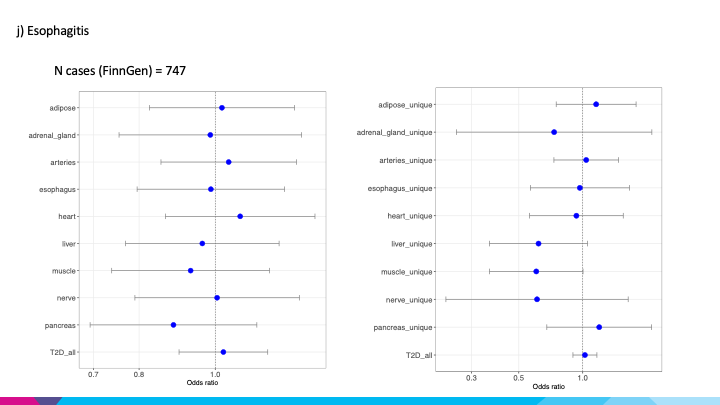
**

**Supplemental Figure 1 a-j.** Results of 2-Sample MR analyses on T2D variants overlapping regulatory elements in tissue of interest (left panel) and 2-Sample MR analysis on T2D variants overlapping unique regulatory elements in tissue of interest (right panel). T2D_all SNP set comprises all 425 lead variants identified in individuals of European ancestry in [7]. The x-axis shows the odds-ratios of the associations, and the y-axis shows the phenotypes tested. Error bars show the 95% CI.

**2a)**
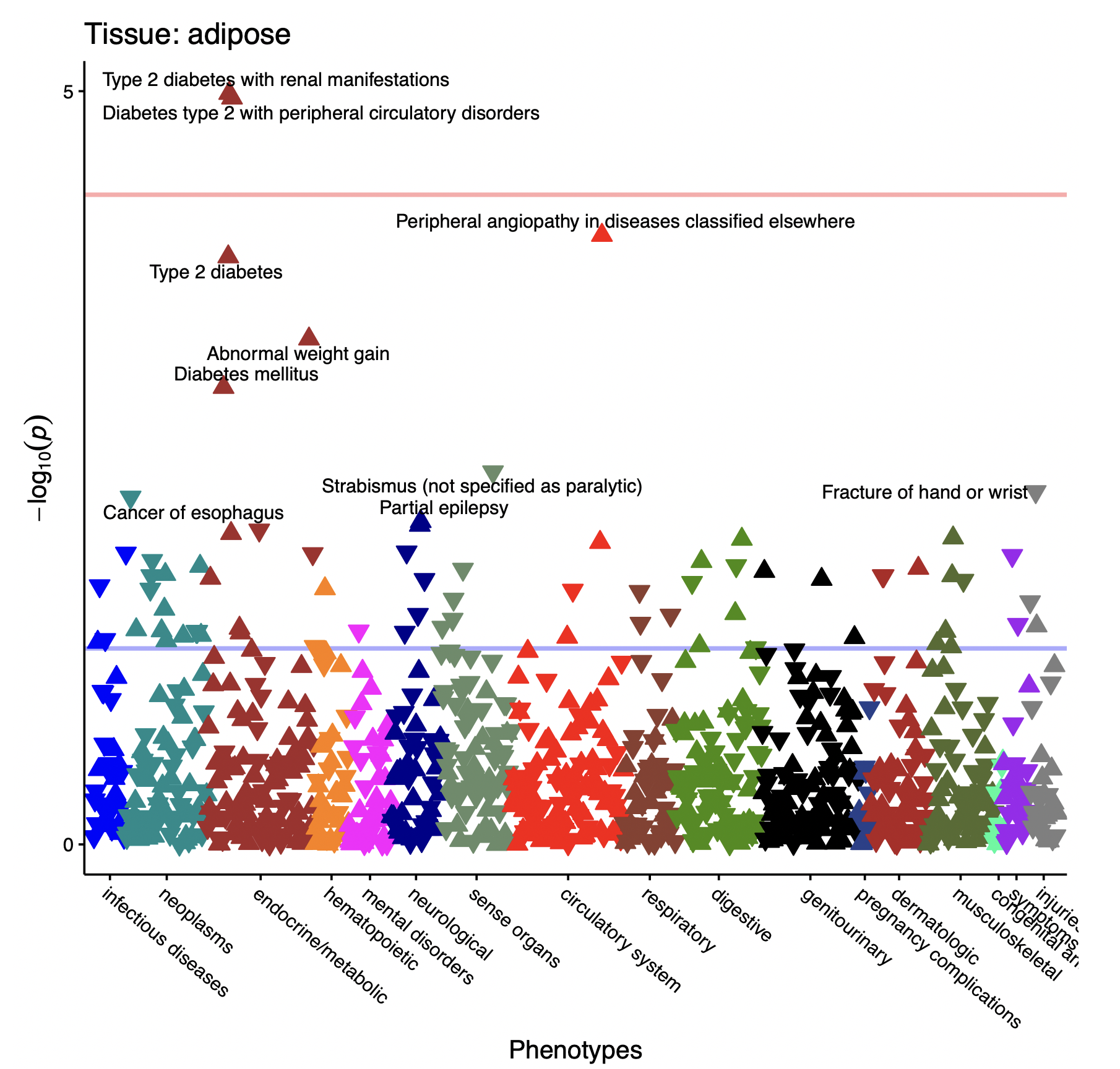


**2b)**
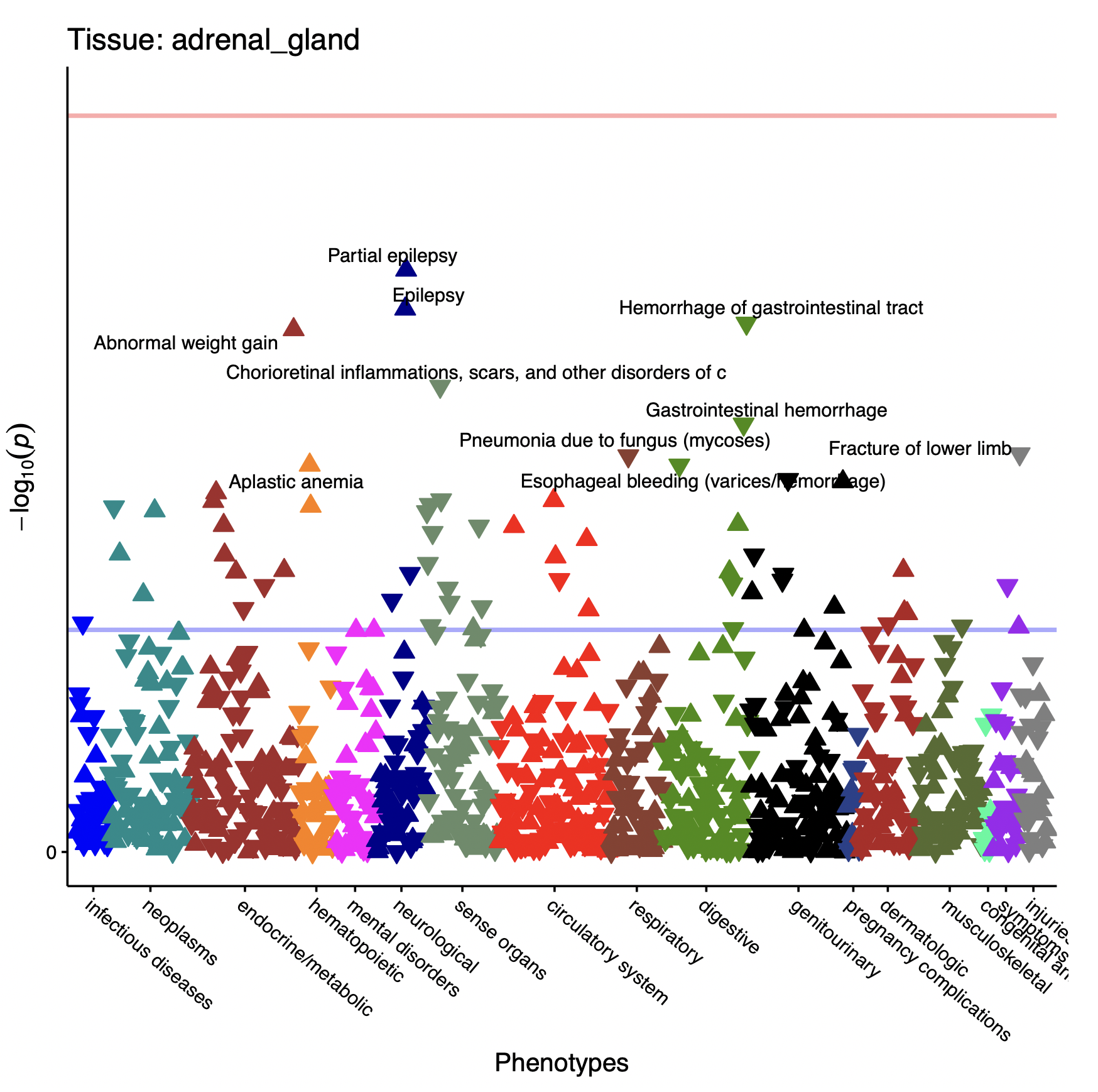


**2c)**
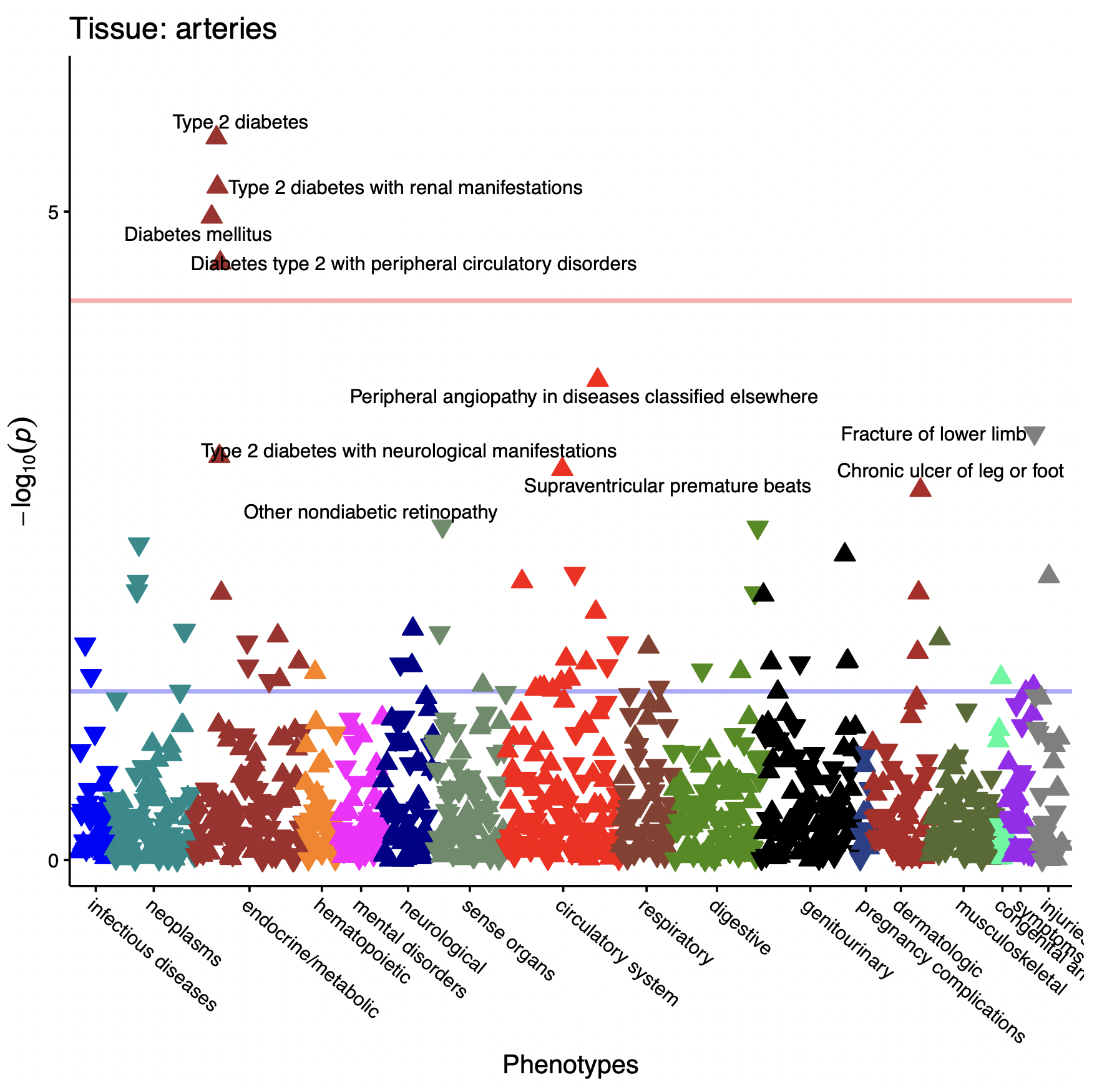


**2d)**
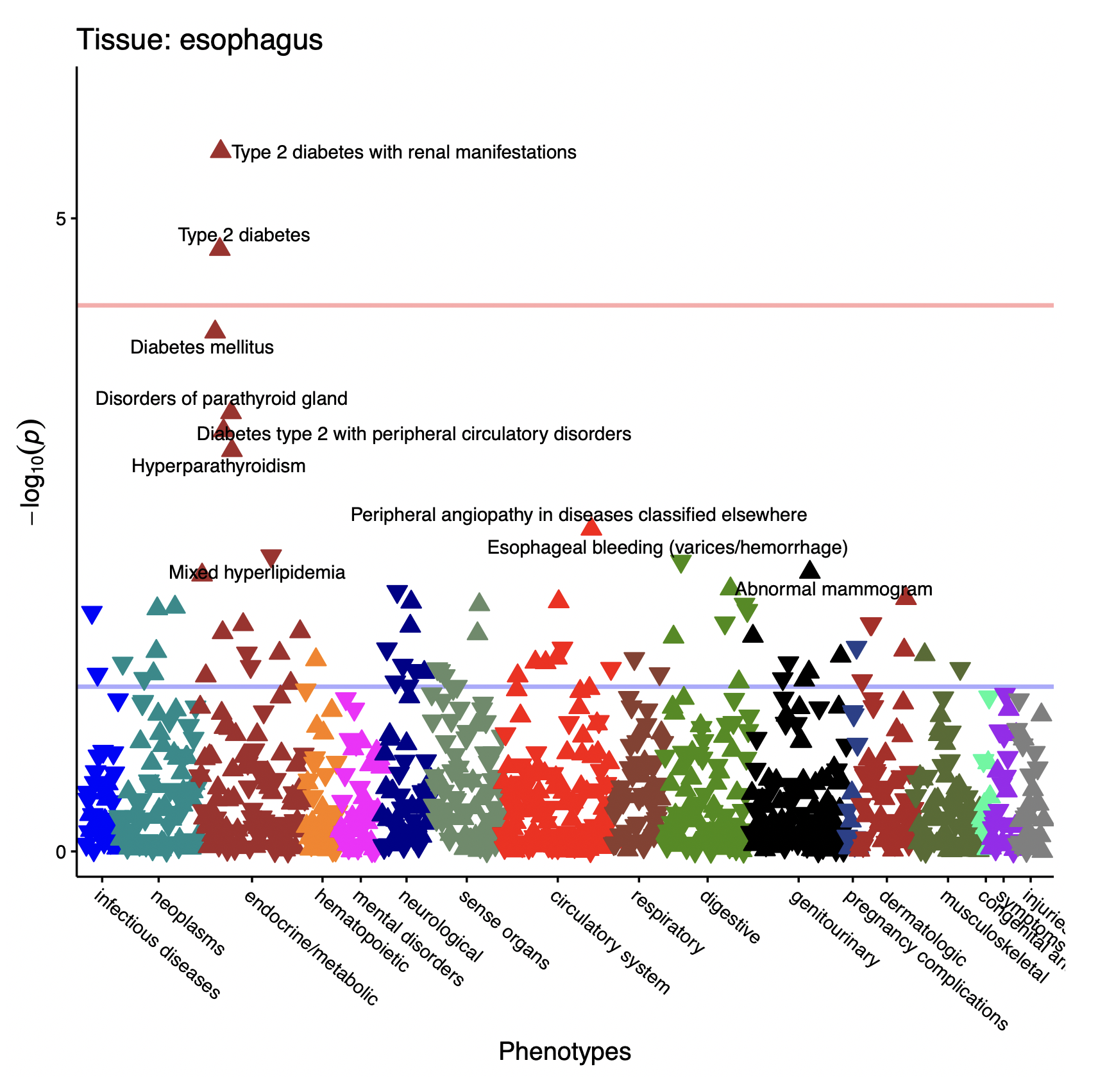


**2e)**
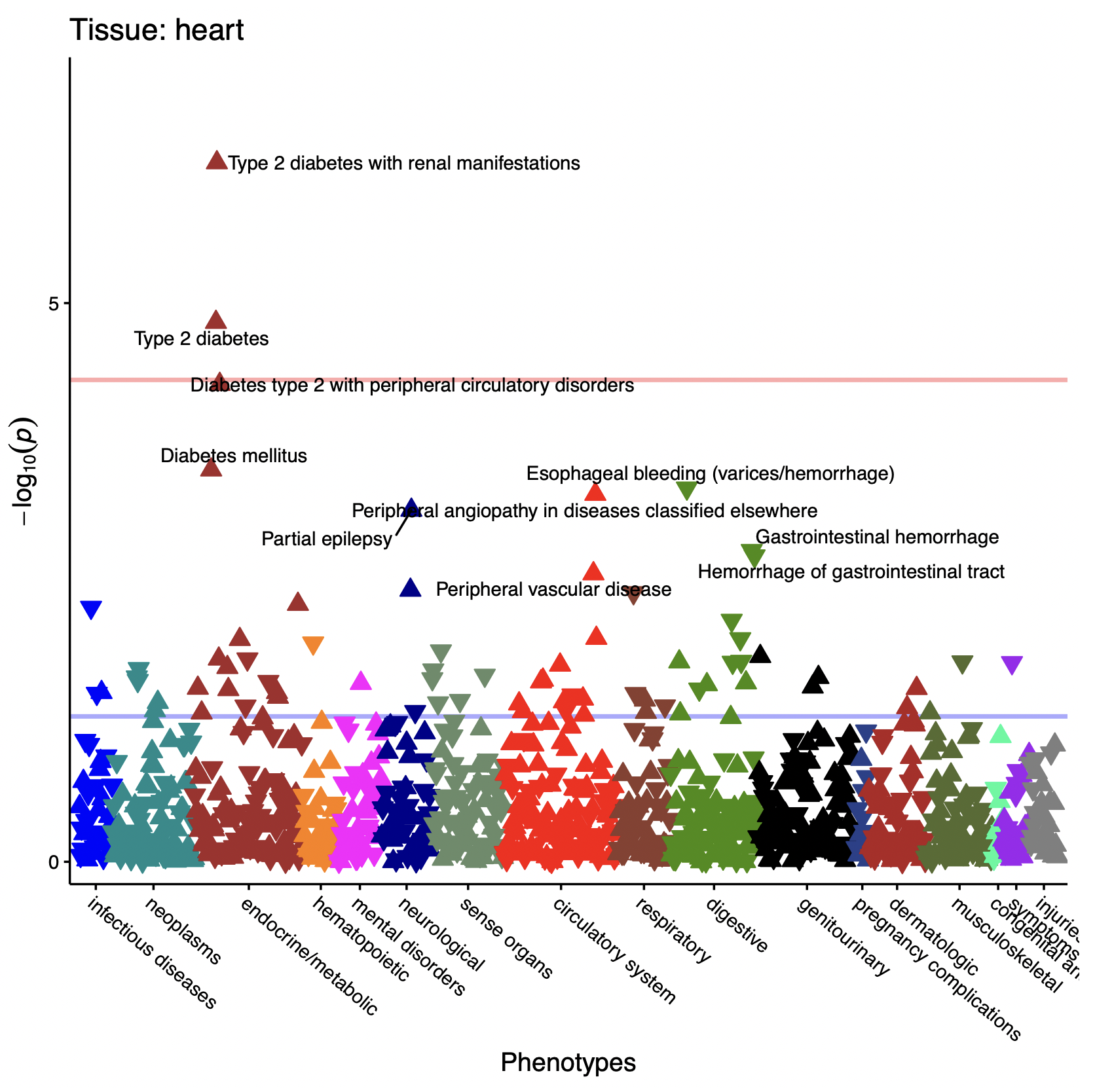


**2f)**
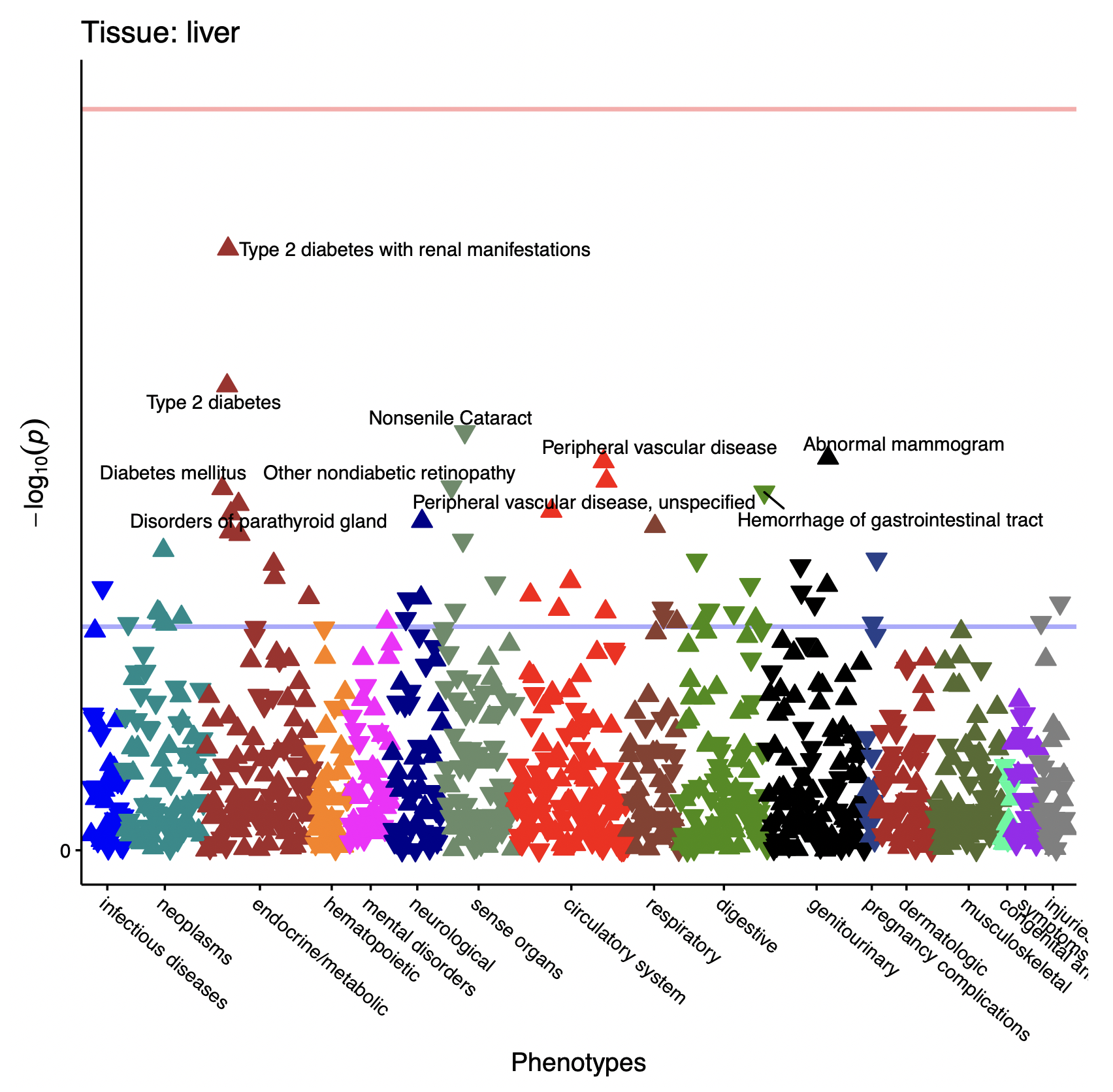


**2g)**
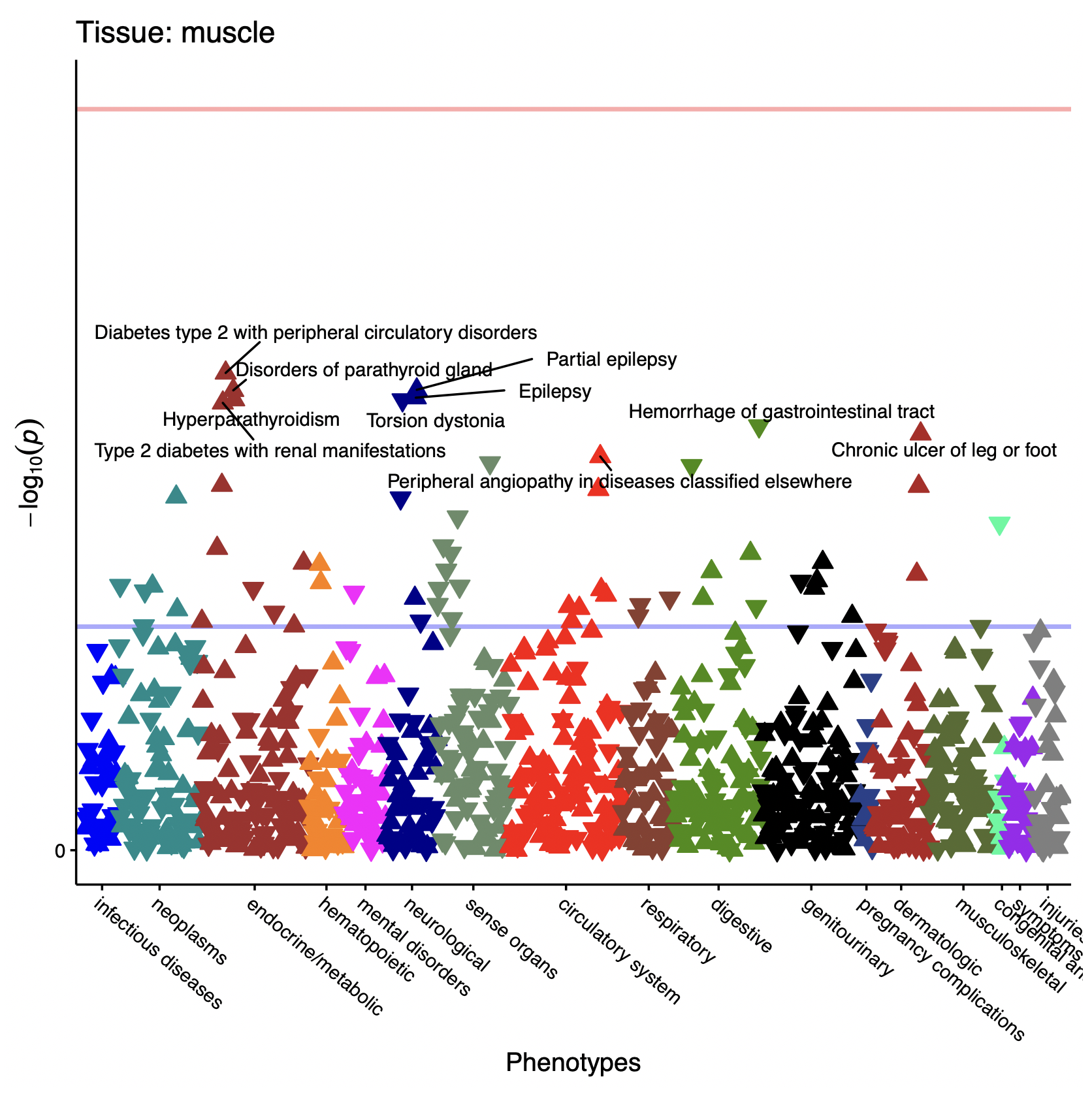


**2h)**
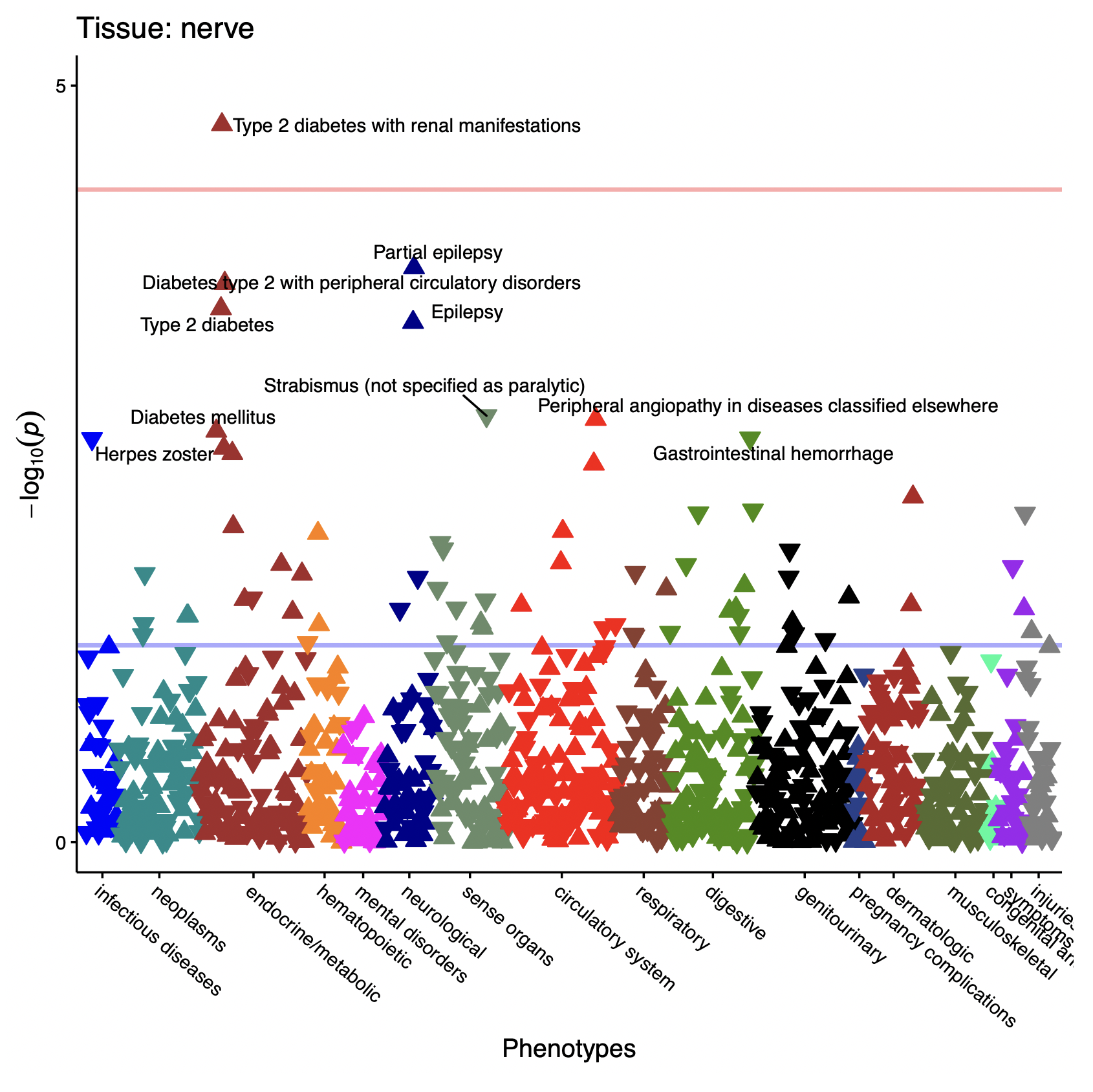


**2i)**
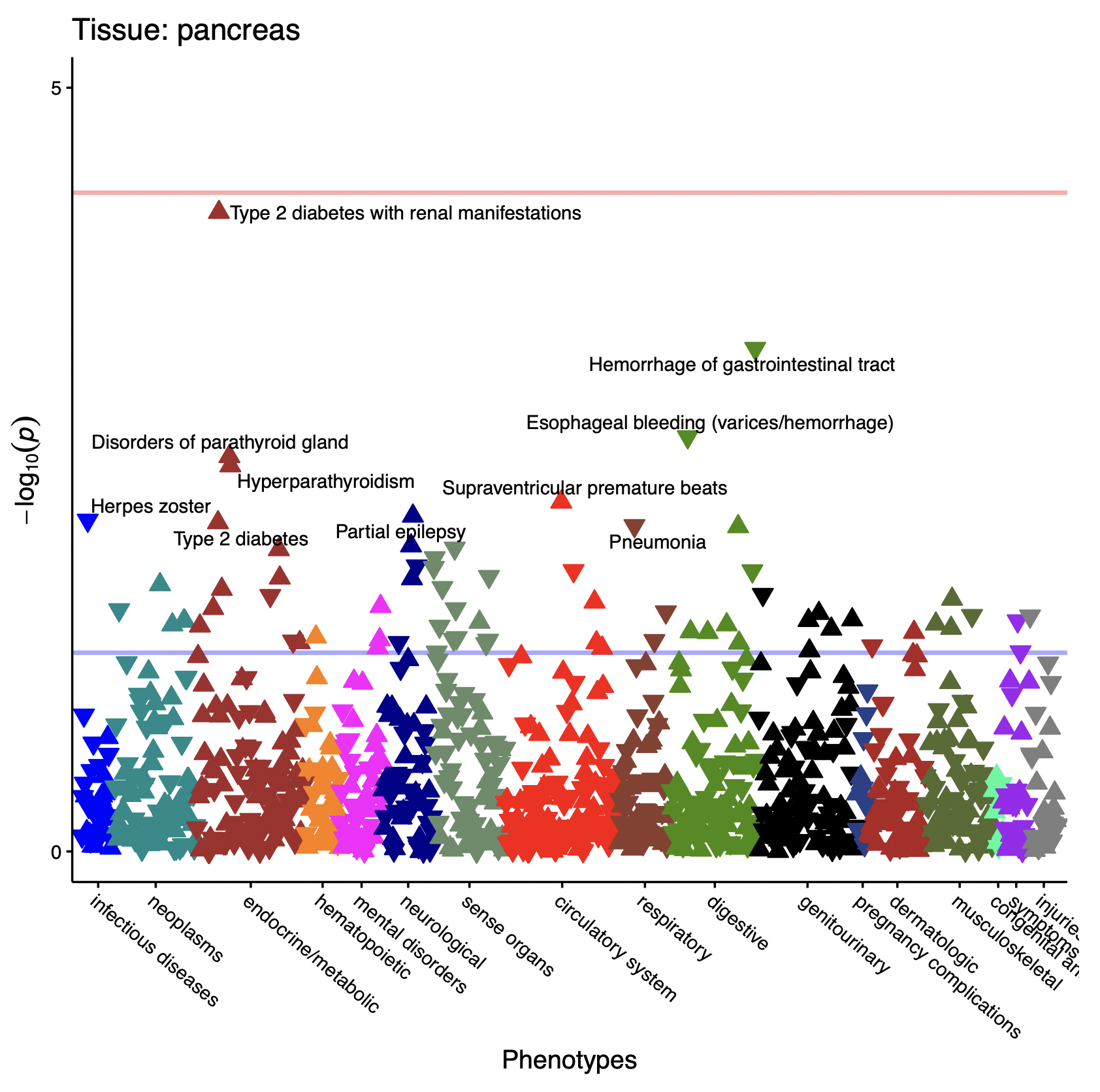


**Supplemental Figure 2:** Association of tissue-grouped SNP sets with multiple phenotypes in the BioMe study for different tissues: **a)** adipose; **b)** adrenal gland; **c)** arteries; **d)** esophagus; **e)** heart; **f)** liver; **g)** muscle; **h)** nerve; **i)** pancreas. The x-axis shows major groups of disease phenotypes, and the y-axis shows the significance of the associations, in -log_10_(p).

**Supplemental Material**

**Supplemental Table 1.** Tissue enrichment analysis of T2D-associated variants on 806 enhancer datasets of Epimap.

**Supplemental Table 2.** Samples from Epimap project whose ChromHMM predictions were used to create a database of high confidence promoters and enhancers.

**Supplemental Table 3.** Results of the 2-Sample MR analysis between each tissue-grouped SNP set and ten secondary outcomes.
